# Impact of Obesity on Atrial Fibrillation Pathogenesis and Treatment Options

**DOI:** 10.1161/JAHA.123.032277

**Published:** 2023-12-29

**Authors:** Rina Sha, Olivia Baines, Abbie Hayes, Katie Tompkins, Manish Kalla, Andrew P. Holmes, Christopher O'Shea, Davor Pavlovic

**Affiliations:** ^1^ Institute of Cardiovascular Sciences, University of Birmingham Birmingham United Kingdom

**Keywords:** antiarrhythmic drugs, atrial fibrillation, epicardial adipose tissue, ion channel, obesity, Atrial Fibrillation, Electrophysiology, Arrhythmias, Catheter Ablation and Implantable Cardioverter-Defibrillator

## Abstract

Atrial fibrillation (AF) is the most common cardiac arrhythmia. AF increases the risk of stroke, heart failure, dementia, and hospitalization. Obesity significantly increases AF risk, both directly and indirectly, through related conditions, like hypertension, diabetes, and heart failure. Obesity‐driven structural and electrical remodeling contribute to AF via several reported mechanisms, including adiposity, inflammation, fibrosis, oxidative stress, ion channel alterations, and autonomic dysfunction. In particular, expanding epicardial adipose tissue during obesity has been suggested as a key driver of AF via paracrine signaling and direct infiltration. Weight loss has been shown to reverse these changes and reduce AF risk and recurrence after ablation. However, studies on how obesity affects pharmacologic or interventional AF treatments are limited. In this review, we discuss mechanisms by which obesity mediates AF and treatment outcomes, aiming to provide insight into obesity‐drug interactions and guide personalized treatment for this patient subgroup.

**Nonstandard Abbreviations and Acronyms**
AAD: antiarrhythmic drug
APD: action potential duration
DOAC: direct oral anticoagulant
EAT: epicardial adipose tissue
ERP: effective refractory period
HFD: high‐fat diet
NLRP3: NACHT, LRR, and PYD domains containing protein 3
POAF: postoperative atrial fibrillation

Atrial fibrillation (AF) is the most common arrhythmia, affecting an estimated 60 million people worldwide.^1^ AF incidence is predicted to increase >60% by 2050.^1^ Several risk factors promote the onset and progression of AF, including age, genetic factors, hypertension, obesity, diabetes, and obstructive sleep apnea.^1^ Epidemiologic studies demonstrate that obesity is the strongest predictor for developing AF following hypertension.^1^, ^2^ Latest data from the World Health Organization report that over half of all adults were overweight or obese.^3^ Genetic predisposition to obesity is related to a higher risk of incident AF, even after adjusting for other traditional risk factors.^4^ The ongoing Framingham Heart Study, initiated in 1948, has shown that obesity increases the risk of developing new‐onset AF by 50%.^5^ A 5‐unit increase in body mass index (BMI) confers a 30% increase in AF incidence^6^. Obesity is also related to the prevalence of several comorbidities, including hypertension, diabetes, coronary artery disease, obstructive sleep apnea, and heart failure, all of which contribute to the development of AF.^7^ With an increasingly overweight population, obesity‐linked AF will continue to progress as a major health burden worldwide.

Obesity‐mediated structural and electrical remodeling both contribute to AF. Ectopic cardiac adipose tissue deposits, particularly epicardial adipose tissue (EAT), appear to play a central role and, hence, provide an attractive target for novel therapies.^8^ Weight loss via lifestyle modification or bariatric surgery demonstrates reversal of obesity‐mediated remodeling and improved AF outcomes.^9^, ^10^

Treatment strategies for AF include rate control (eg, β‐blockers), rhythm control (eg, antiarrhythmic drugs and ablation), and stroke prevention with anticoagulation. Obesity can alter the efficacy of these treatments. For example, patients with AF had a 13% higher risk of experiencing AF recurrence after ablation surgery for every 5‐unit increase in BMI^6^. Further studies suggest that obesity alters the efficacy and/or pharamacokinetics of antiarrhythmic drugs (AADs), β‐blockers, and anticoagulation agents.^11^, ^12^, ^13^, ^14^, ^15^ However, for all these treatments, there is an incomplete understanding of the mechanistic relationship between obesity and treatment, as there is for AF onset and progression. In this review, we provide a critical narrative on obesity, its role in AF pathogenesis, and implications for therapy.

## PATHOGENESIS OF AF IN OBESITY

Obesity leads to elevated arterial, left ventricular, and left atrial (LA) filling pressures, driving diastolic dysfunction.^7^, ^16^ Increased circulating blood volume during obesity leads to higher LA and left ventricular workload, triggering chamber dilatation and myocyte hypertrophy, to maintain a larger cardiac output.^17^ Therefore, obesity is strongly associated with LA enlargement, an important precursor of AF.^18^ The mechanisms for this are unclear, and possibly distinct and more complex from those mediated by hypertension‐driven enlargement. Hearts of obese individuals and in preclinical models also show significant accumulation of lipid deposition in the myocardium and pericardium.^19^ Evidence of increased cardiac fibrosis has been reported in obese patients and animal models,^18^, ^20^ although direct causation has not been clearly demonstrated. Below, we discuss mechanisms driving AF in obesity.

## ADIPOSE TISSUE AND CARDIOVASCULAR FUNCTION

Adipose tissue is one of the largest endocrine organs in the body and can be classified as visceral or subcutaneous fat. According to cellular origins and molecular features, mesenchymal stem cells can differentiate into white, brown, and brite or beige adipocytes.^21^ White adipose tissue specializes in energy and triglyceride storage.^21^ Brown adipose tissue adipocytes contain more mitochondria to perform thermogenesis and oxidation, maintaining body temperature.^21^ Beige adipose tissue is regarded as an intermediary phenotype.

Brown‐to‐white adipocyte transdifferentiation correlates with increased oxidative stress and has been reported in EAT of patients with coronary artery disease.^22^ It is unclear whether this *whitening* phenotype transition contributes to obesity‐mediated AF. Peroxisome proliferator‐activated receptors regulate glucose and lipid metabolism. Studies have reported downregulated peroxisome proliferator‐activated receptor‐γ coactivator 1‐α in EAT of patients with coronary artery disease,^22^ presenting a possible target for reversing metabolic dysfunction in obesity. Future studies will need to evaluate both adipocyte phenotype heterogeneity (brown/white adipocyte ratio) and tissue distribution in obesity‐mediated AF.

## MECHANISMS OF OBESITY‐MEDIATED AF

**Figure 1:**
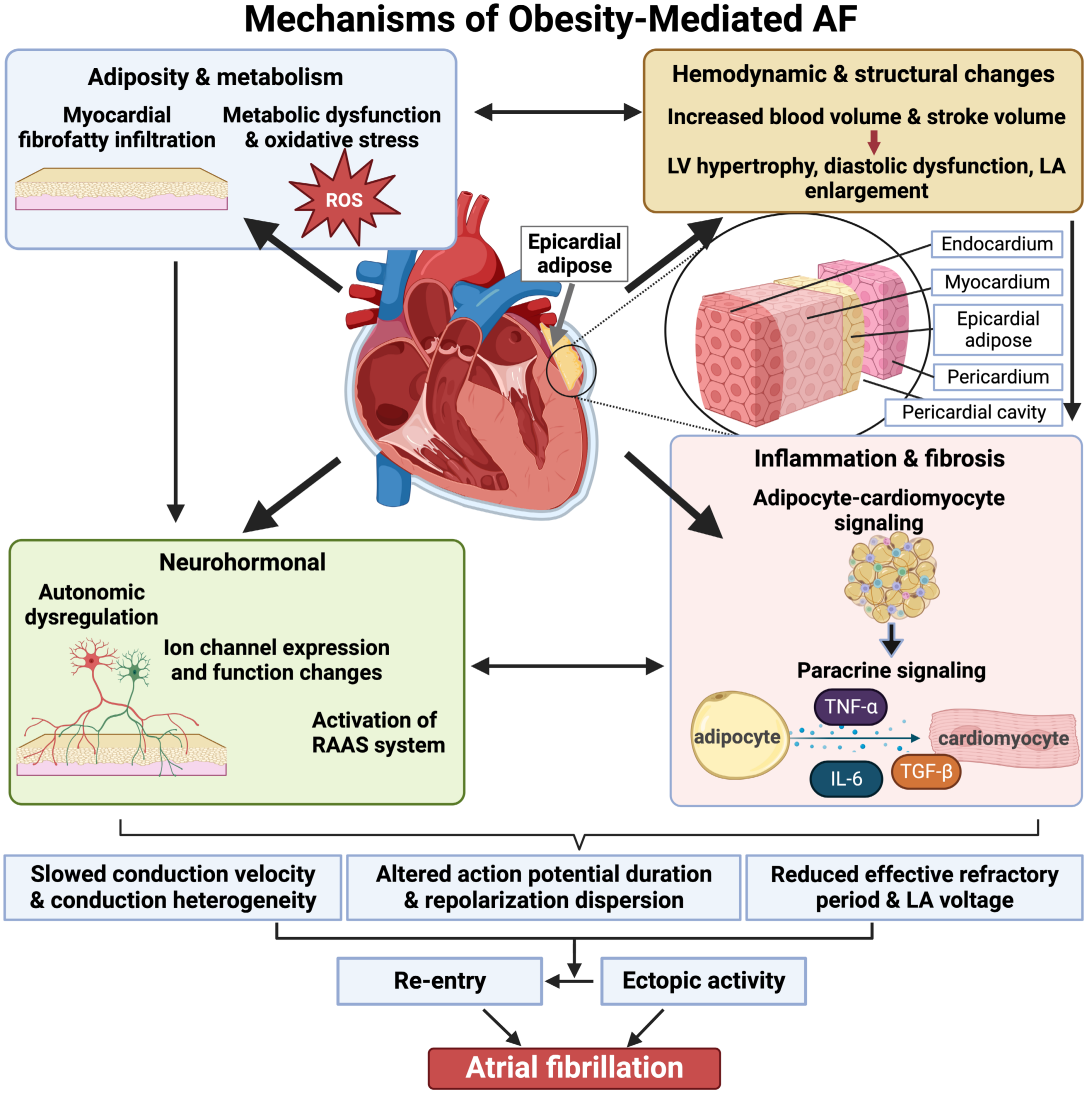
Mechanisms of obesity‐mediated atrial fibrillation (AF). Obesity‐mediated AF is multifactorial, encompassing hemodynamic, structural, and electrical remodeling, adiposity, metabolic dysfunction, inflammation, and neurohormonal changes. Epicardial adipose tissue accumulates during obesity and can mediate structural and electrical remodeling. Subsequent conduction and repolarization abnormalities lead to increased ectopic activity and wavelength reentry, causing AF. IL indicates interleukin; LA, left atrial; LV, left ventricular; RAAS, renin‐angiotensin‐aldosterone system; ROS, reactive oxygen species; TGF‐β, transforming growth factor‐β; and TNF‐α, tumor necrosis factor‐α. Figure created with BioRender.com.

Several mechanisms have been suggested in obesity‐driven AF, including cardiac adiposity, inflammation, fibrosis, oxidative stress, ion channel remodeling, and autonomic dysfunction (Figure 1). These mechanisms promote the development of AF triggers and substrate, ultimately leading to increased AF vulnerability.

## THE ROLE OF EAT IN AF

EAT, a type of visceral fat, is located between the myocardium and visceral pericardium. It is originally derived from brown adipose tissue, performing thermogenic functions during neonatal development, and then transitioning to a mixed, beige‐like phenotype by adulthood for cardiac energy storage purposes.^8^ EAT predominantly consists of adipocytes; however, ganglionic plexi, smooth muscle, fibroblasts, stem cells, endothelial cells, and immune cells also coexist.^8^ In normal physiology, the EAT performs cardioprotective lipogenesis and fatty acid metabolism,^23^ and releases adipokines (eg, adiponectin) to regulate oxidative stress and inflammation.^24^ Uniquely, the myocardium and EAT are contiguous, sharing the same microcirculation and therefore promoting adipocyte‐cardiomyocyte paracrine signaling.^8^

Population studies demonstrate EAT volume is independently associated with AF occurrence, severity, and recurrence, and therefore may mediate the link between obesity and AF.^25^, ^26^ This association was stronger than those detected for measures of abdominal or overall adipose tissue (including BMI), suggesting a unique role for EAT.^26^ Increased LA EAT volume also independently correlated with greater LA fibrosis and dilatation, which are predictors for AF.^19^ Several preclinical studies demonstrate a significant role for EAT in AF pathogenesis (eg, via paracrine signaling) (see subsections Inflammation, Fibrosis, and Oxidative Stress; Neurohormonal Changes; and Electrical Remodeling).

Obese patients with AF show an increase in EAT volume, especially around the posterior LA and the atrioventricular groove.^19^ Moreover, human‐induced pluripotent stem cell–derived cardiomyocytes from patients with AF and incubated with EAT‐extracted extracellular vesicles from patients with AF demonstrated sustained reentry.^27^ Authors additionally showed EAT to secrete proinflammatory, profibrotic, and proarrhythmic substances,^27^ highlighting a major pathologic role of EAT in obesity‐mediated AF.

## INFLAMMATION, FIBROSIS, AND OXIDATIVE STRESS

**Figure 2:**
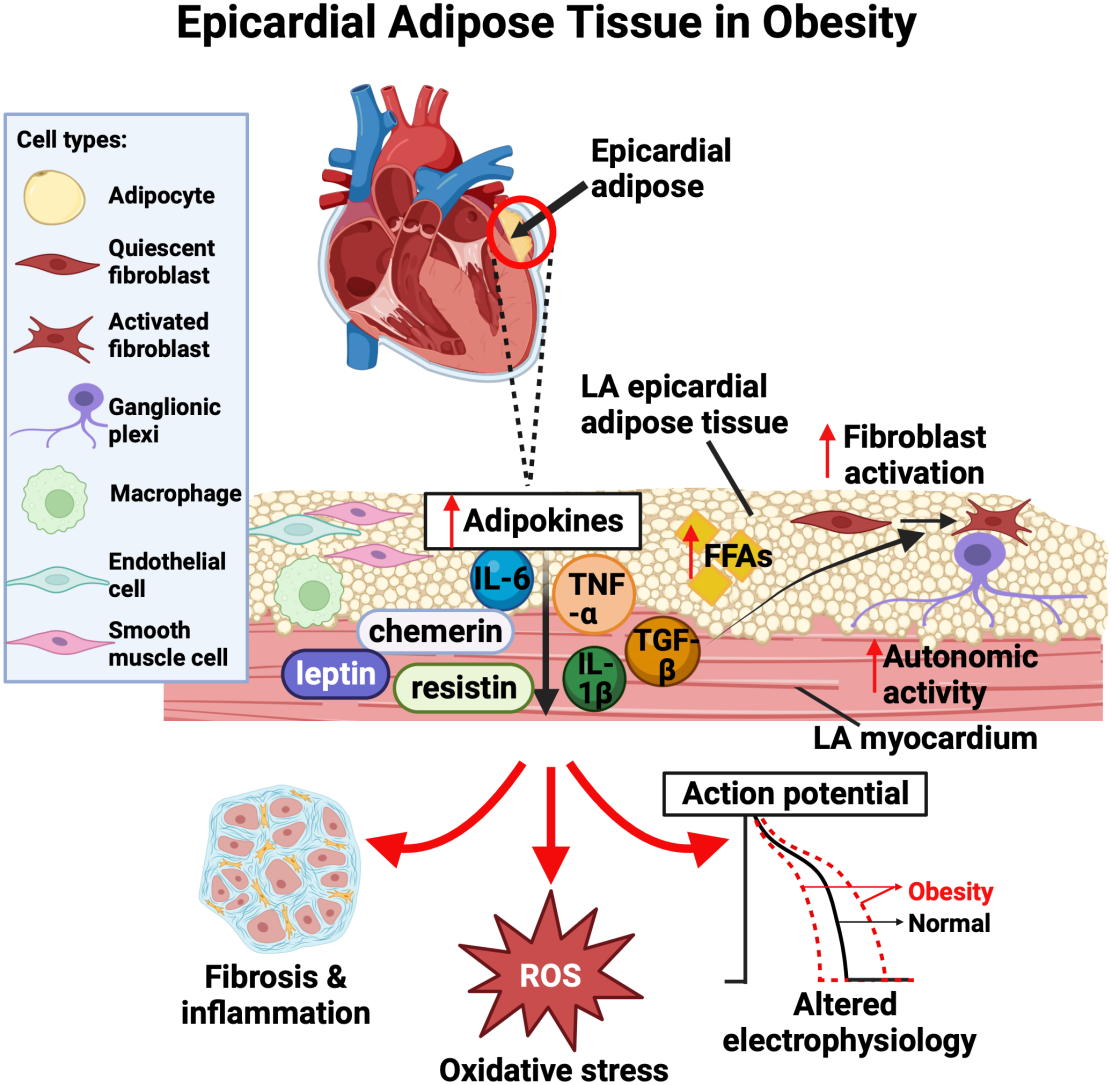
The effect of left atrial (LA) epicardial adipose tissue (EAT) on myocardial structure and function in obesity. Obesity causes EAT expansion, particularly around the posterior LA, and dysfunction. EAT is composed of adipocytes, fibroblasts, ganglionic plexi, macrophages, endothelial cells, and smooth muscle cells. In obesity, the expression and secretion of certain adipokines in EAT are increased. Alongside excess free fatty acids (FFAs) and ganglionic plexi hyperactivity‐induced autonomic dysfunction, this leads to LA fibrosis, inflammation, oxidative stress, and altered atrial electrophysiology. IL indicates interleukin; ROS, reactive oxygen species; TGF‐β, transforming growth factor‐β; and TNF‐α, tumor necrosis factor‐α. Figure created with BioRender.com.

The link between inflammation and AF is well established. Patients with AF show upregulated inflammatory cytokines, including interleukin‐6, interleukin‐1β, CRP (C‐reactive protein), and tumor necrosis factor (TNF)‐α.^28^, ^29^ All but interleukin‐1β were also shown to be upregulated in otherwise healthy obese women, suggesting that adipose tissue contributes to systemic inflammation.^30^ Inflammatory cytokines were also shown to increase oxidative stress, collagen deposition, and abnormal calcium handling, resulting in tissue damage, local fibrosis, and altered electrophysiology of the atrial tissue.^24^ For example, interleukin‐6 stimulates differentiation of naive T cells to effector type 17 helper T cells,^31^ leading to interleukin‐17–mediated myocardial fibrosis.^32^ EAT can release proinflammatory cytokines and adipokines (Figure 2), including chemerin and resistin, associated with atrial remodeling.^33^, ^34^ In addition, enhanced atrial activity of the NLRP3 (comprised of central nucleotide‐binding and oligomerization (NACHT) domain, C‐terminal leucine‐rich repeat (LRR) domain, and N‐terminal effector pyrin domain (PYD)–containing protein 3) inflammasome was reported in obese patients.^35^ Homozygous NLRP3 knockout high‐fat diet (HFD)–induced obese mice exhibited reduced AF susceptibility compared with HFD controls, suggesting obesity‐mediated increased NLRP3 activity promotes AF.^35^ The NLRP3 inflammasome is known to secrete proinflammatory cytokine interleukin‐1β through caspase‐1 cleavage, which is associated with macrophage polarization and subsequent electrical remodeling in right atrial appendage tissue of patients with AF.^36^ NLRP3 activity was also shown to upregulate ultrarapid delayed‐rectifier potassium channel,^35^ which contributes to AF substrate development through shortened action potential duration (APD) and effective refractory period (ERP) (see section Electrical Remodeling).

Atrial enlargement and fibrosis are arguably the most reliable predictors for AF.^37^, ^38^ Interstitial fibrosis is characterized by enhanced cardiac fibroblast activation, proliferation, and extracellular matrix deposition. Altered fibroblast properties and ratio of fibroblasts/cardiomyocytes can disrupt cardiac fibroblast‐cardiomyocyte electrical coupling and the source‐sink relationship, which usually protects against triggered activity propagating into surrounding tissue.^18^, ^39^ Therefore, interstitial fibrosis promotes the development of triggered activity and unidirectional block, increasing vulnerability to reentry.^18^ EAT can release a myriad of profibrotic adipocytokines, including those in the transforming growth factor‐β superfamily, promoting atrial fibroblast activation and subsequently interstitial fibrosis.^18^ Moreover, patients with AF and HFD‐induced obese mice exhibit enhanced fibroblast‐activating factor cadherin‐11 expression in the LA, contributing to atrial fibrosis.^20^ In support of this, HFD in homozygous cadherin‐11 knockout mice did not induce atrial dilatation, inflammation, or fibrosis, as observed in HFD obese wild‐type mice.

Somewhat surprisingly, fibrosis and EAT volume measured in right atrial samples of patients with permanent AF were inversely correlated.^40^ Nevertheless, the prevalence of fibrofatty infiltrates was significantly higher in patients with permanent AF than patients with paroxysmal AF.^40^ This finding was confirmed in a persistent AF sheep model without confounding morbidities.^40^ Future studies should investigate the established correlation between epicardial fibrosis and AF duration,^40^ focusing on understanding the mechanisms driving fibrotic remodeling in subepicardial fatty infiltrates in obesity‐mediated AF.

Obesity and EAT have also been associated with higher reactive oxygen species production, oxidative stress, and impaired autophagy.^24^, ^41^, ^42^ Administering mitochondrial antioxidants to HFD mice reversed electrical and structural remodeling, alleviating AF burden.^41^

## NEUROHORMONAL CHANGES

EAT hosts numerous ganglionic plexi, which are important in regulating cardiac autonomic function.^43^ Ganglionic plexi integrate both vagal and sympathetic signals to regulate changes in heart rate and contractility.^44^ In AF, ganglionic plexi hyperactivity and increased atrial sympathetic neuron density drive autonomic dysfunction, shortened ERP, and amplified magnitude of calcium transients.^44^, ^45^ Vagal activation increases atrial ERP heterogeneity, perpetuating arrhythmogenesis.^46^ Sympathetic and vagal nerves are adjacent in ganglionic plexi and have opposing functions. However, both sympathetic and parasympathetic influences have been suggested to contribute to AF initiation and maintenance. Focal firing associated with APD shortening was shown to be dependent on both sympathetic and parasympathetic activity in isolated superfused canine pulmonary veins.^47^

Obesity also drives autonomic nervous system imbalance. Augmented sympathetic activation is observed in obese patients,^48^ induced by hypoxemia,^49^ hyperleptinemia,^50^ and insulin resistance.^48^ HFD‐induced obese rats exhibit significant right ventricular sympathetic hyperinnervation and outgrowth alongside reduced basal atrial acetylcholine release, associated with greater AF inducibility.^51^ Randomized, double‐blinded clinical trials have examined the advantages of autonomic activity suppression in patients with preoperative paroxysmal AF undergoing coronary artery bypass graft surgery.^52^, ^53^ Targeted autonomic denervation of ganglionic plexi embedded in EAT, using site‐specific botulinum toxin injection during or immediately after surgery, reduced AF recurrence. Adipocytes can also produce angiotensinogen, angiotensin, and angiotensin‐converting enzyme supplementary to their systemic production, thereby locally and systemically activating renin‐angiotensin‐aldosterone system.^54^ TNF‐α, released by EAT in obesity, was also demonstrated to enhance adipocyte angiotensinogen protein expression and angiotensin II secretion, providing further evidence of the link between obesity and renin‐angiotensin‐aldosterone system activation.^54^ Combined with EAT sympathetic hyperactivity and release of inflammatory adipokine leptin, this drives obesity‐mediated renin‐angiotensin‐aldosterone system activation and subsequent myocardial remodeling.^48^, ^50^ Angiotensin II, a key element of renin‐angiotensin‐aldosterone system, also depresses metalloproteinase‐1 expression and induces collagen type 1 synthesis in cardiac fibroblasts,^55^ causing fibrosis.

## ELECTRICAL REMODELING

**Figure 3:**
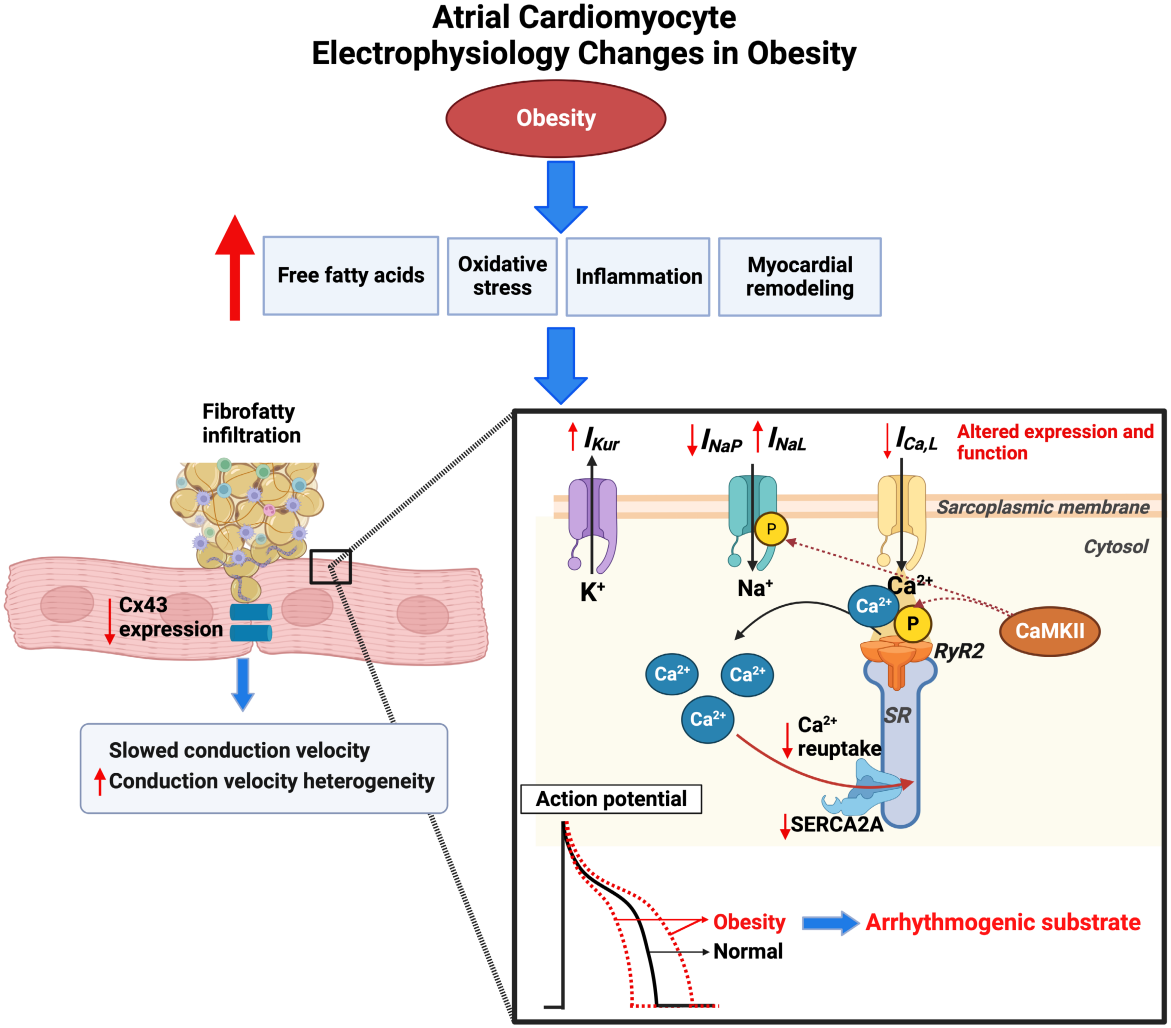
The effect of obesity on atrial cardiomyocyte electrophysiology. Obesity can induce excess free fatty acids, oxidative stress, inflammation, and myocardial remodeling. Electrical remodeling is subsequently triggered, through mechanisms not fully elucidated, causing altered expression and function of atrial ion channels and gap junctions. Subsequent abnormal atrial conduction and repolarization properties characterize arrhythmogenic substrate, increasing risk of atrial fibrillation. CaMKII indicates calmodulin kinase 2; Cx43, connexin 43; I_Ca,L_, L‐type calcium current; I_Kur_, ultrarapid potassium current; I_Na,L_, late sodium current; I_Na,P_, peak sodium current; P, phosphorylation; RyR2, ryanodine 2 receptor; SERCA2A, sarcoplasmic/endoplasmic reticulum Ca^2+^‐ATPase 2a; and SR, sarcoplasmic reticulum. Figure created with BioRender.com.

Robust evidence supports the concept that obesity leads to multiple electrophysiological changes, via direct effects of fatty infiltrate on conduction, via paracrine factors released from EAT or activated fibroblasts, via cardiomyocyte‐fibroblast coupling, via stretch‐dependent mechanisms, and via electrical remodeling (Figure 3), all leading to slowed and heterogeneous conduction and shortened ERP.^16^, ^18^, ^40^, ^56^ Shortened ERP reduces cardiac wavelength and promotes reentry, a key mechanism of AF onset and maintenance.^57^

Adipocytes from EAT have been shown to directly infiltrate the myocardium of the LA posterior wall in HFD sheep.^18^, ^40^ Fatty infiltrates in the myocardium correlate with regional low endocardial voltage. Myocardial fat infiltration by abundant EAT may cause a physical conduction barrier, disrupting inter‐cardiomyocyte signaling, enhancing paracrine signaling, or both, thus leading to conduction abnormalities.^18^, ^56^ EAT‐derived adipocytokines also directly shorten APD and promote sustained rotors in human‐induced pluripotent stem cell–derived cardiomyocytes.^27^ In rat ventricular myocytes treated with leptin, an adipocytokine expressed in EAT, there is upregulation of voltage‐gated potassium (Kv) 4.2 and Kv4.3 channel subunits,^58^ thus leading to larger fast transient outward potassium current amplitudes and shortened APD.

Enhanced cardiomyocyte‐fibroblast electrical coupling attributable to atrial fibrosis may slow conduction velocity and increase conduction heterogeneity, contributing to AF in obesity.^18^, ^39^ In an obesity sheep model, alterations in connexin‐43 gap junction protein expression have also been observed,^56^ highlighting an additional mechanism of conduction slowing.

Obesity‐induced elevated LA pressure and subsequent atrial stretch reduce atrial ERP and slow atrial conduction velocity, partly via a reduction in cellular excitability by the opening of stretch‐activated channels.^36^ Transient atrial stretch induced in isolated guinea pig hearts using a fluid‐filled intra‐atrial balloon catheter was also associated with early after depolarizations, recorded on the LA epicardium.^59^

Ion channel remodeling has been demonstrated by studies in both patients with AF^60^ and animal models of AF.^41^ Aromolaran et al^61^ found increased delayed rectifier potassium current in guinea pigs with HFD, resulting in shortened APD. Fatty acid–treated HEK293 cells, transiently expressing either rapid or slow potassium channel subunits, were used to further model the effect of obesity‐related specific fatty acids on different potassium current components. Saturated palmitic acid and monosaturated oleic acid treatment augmented and depressed, respectively, rapid maximal potassium current in HEK293 cells expressing human *ether‐a‐go‐go‐*related gene (*hERG*) 1a and 1b. Similarly, palmitic acid showed a greater increase in slow maximal potassium currents in HEK293 cells expressing KCNQ1 (potassium voltage‐gated channel subfamily Q member 1) and KCNE1 (potassium voltage‐gated channel subfamily E regulatory subunit 1), compared with oleic acid treatment. In addition, obesity‐mediated downregulation of voltage‐gated sodium channel and voltage‐gated calcium channel expression and increased ultrarapid delayed‐rectifier potassium channel expression were dependent on mitochondrial‐derived reactive oxygen species.^41^ Ion channel remodeling resulted in reduced peak sodium current, reduced L‐type calcium current, and increased ultrarapid potassium current.^41^ These alterations are likely to account for the shortened APD and reduced conduction velocity. Obesity‐induced oxidative stress was also associated with oxidation of ventricular tissue ryanodine 2 receptors.^62^ Consequent calcium ion leak from the sarcoplasmic reticulum is postulated to activate calmodulin kinase II in a positive feedback loop. In the atria, activated calmodulin kinase II hyperphosphorylates the α subunit of voltage‐gated sodium channel at Ser^571^, driving late sodium current and proarrhythmic afterdepolarizations.^13^, ^63^ Increased protein expression of phosphorylated calmodulin kinase II and phosphorylated ryanodine 2 receptors was also shown in HFD mice, which was associated with reduced L‐type calcium channel protein expression.^64^ Moreover, all these changes were significantly attenuated in mice treated with calmodulin kinase II inhibitor, suggesting calmodulin kinase II is an important upstream mediator in obesity‐mediated calcium handling dysfunction. TNF, a proinflammatory cytokine upregulated in obesity and AF, has been proven to increase the vulnerability to AF and atrial remodeling in animal models.^24^, ^65^ TNF‐α was reported to induce abnormal calcium ion handling by reducing SERCA2A (sarcoplasmic/endoplasmic reticulum Ca^2+^ ATPase 2a) expression in TNF‐α–treated rabbit pulmonary vein cardiomyocytes, thus leading to decreased calcium transient amplitude and prolonged Ca^2+^ transient decay.^65^ In addition, increased sodium‐calcium exchanger current was observed, likely compensating for reduced SERCA2A expression and Ca^2+^ removal, which can trigger delayed afterdepolarizations.^65^ Transgenic mice models also indicate that TNF‐α facilitates significant atrial hypertrophy, fibrosis, contractile failure, and AF inducibility.^24^, ^65^ Moreover, the effects of TNF‐α on atrial and ventricular cardiomyocyte electrophysiology are reported to be sex specific, demonstrating a greater impact in males.^24^

## THE EFFECT OF WEIGHT REDUCTION ON AF

In clinical trials, weight reduction has been shown to improve outcomes of AF ablation by reversing structural and electrical remodeling.^9^, ^66^ Bariatric surgery reduced the risk of new‐onset AF and AF recurrence after ablation.^10^, ^56^ Bariatric surgery also lowered insulin resistance, systolic blood pressure, and EAT volume, associated with reduced AF recurrence.^10^

An observational study demonstrated weight loss, alongside other risk factor modification strategies, reversed structural changes in obese patients with AF.^66^ In a sustained obesity sheep model, a 30% weight reduction correlated with decreased LA EAT volume, myocardial fat filtration, inflammation, normalization of connexin 43 expression, and reversal of fibrosis.^56^ Weight loss–associated reduced transforming growth factor‐β1 and endothelin receptor‐B expression is hypothesized to enable collagen reabsorption and reversal of cardiac fibrosis, consequently reverting obesity‐induced electrical and structural remodeling.^56^ Indeed, weight loss reduced conduction abnormalities and normalized atrial refractoriness, increasing ERP compared with control sheep.^56^ Furthermore, structural remodeling was also reversed with weight loss, including decreased atrial fibrosis and LA pressure.

## OBESITY PARADOX IN AF

Studies investigating the relationship between obesity and major adverse events in patients with AF have shown controversial results. Badheka et al^67^ reported that overweight and obese patients with AF demonstrated a reduced risk of both cardiovascular and all‐cause death. This phenomenon is known as the *obesity paradox*. However, another study including 3135 patients with AF with a median follow‐up of 4.9 years showed that obesity was related to a higher risk of major adverse events and death.^68^ After these initial investigations, more studies have been dedicated to answering whether an obesity paradox exists in AF. Presently, there is no unified conclusion. The ENGAGE AF‐TIMI 48 (Effective Anticoagulation With Factor Xa Next Generation in Atrial Fibrillation–Thrombolysis in Myocardial Infarction 48) trial revealed that overweight and obese patients with AF had lower risk of stroke/systemic embolism and mortality.^69^ The conclusion was challenged by a meta‐analysis, whose authors concluded that the risk for all‐cause and cardiovascular death in obese patients was similar with that in normal‐weight patients.^70^ This controversy also extends to bleeding outcomes. A recent systematic review, including data from randomized control trials, observational studies, and meta‐analyses, reported that overweight and obese patients with AF had a lower risk of major bleeding events than healthy‐weight contemporaries.^71^ The result opposed the recent ENGAGE AF‐TIMI 48 trial, which showed that the risk of all bleeding outcomes increased with every 5‐kg/m^2^ BMI increase.^69^

The reason for these controversial results is unclear. Studies from randomized controlled trial subgroups generally support the obesity paradox, whereas data from observational/population‐based cohort studies are more contradictory.^72^ One possible (but not fully explored) explanation for the obesity paradox is that overweight and obese patients are generally treated earlier and more intensively, and have more rigorous follow‐up. It has also been postulated that higher BMI could reduce death in patients with AF, as it provides greater metabolic reserve.^73^ Therefore, overweight and obese patients may be more able to endure the increased catabolic stress with disease development,^73^ although this is yet to be directly tested.

Statistical artifacts may also explain the obesity paradox, such as underpowered analyses and unreported selection confounders/bias. In addition, the sensitivity of BMI as a measure of obesity, widely used across clinical trials, is limited. BMI does not consider the distribution or composition of adiposity, which are important predictors of AF risk.^26^ Arguably, EAT volume may be the most superior predictor of AF risk.^26^ Further prospective studies with longer follow‐up are needed to clarify the obesity paradox, which more accurately distinguish obesity subtypes within cohorts, using fat distribution measures, such as EAT volume.

Despite the existence of the obesity paradox, the evidence that weight loss directly leads to AF burden reduction and substrate reversal in overweight and obese patients with AF is clear.^9^, ^66^ Therefore, lifestyle management and weight reduction are still important for overweight and obese patients.

## THE EFFECT OF OBESITY ON AF TREATMENT

**Figure 4:**
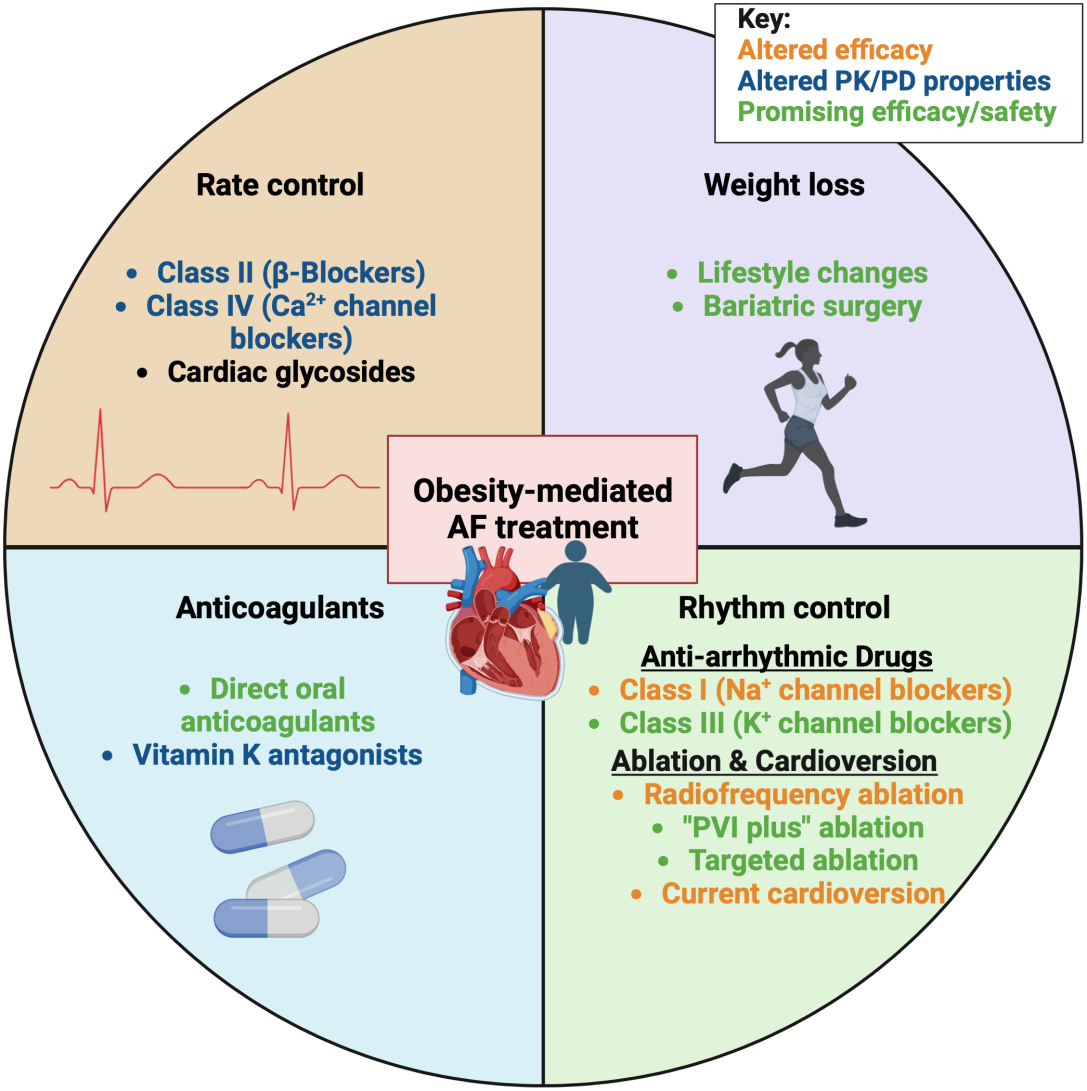
The effect of obesity on current atrial fibrillation (AF) treatments and potential recommendations for treatment of patients with obesity‐mediated AF. Existing clinically used AF treatments with *altered efficacy* in obesity are shown in orange, *altered pharmacokinetic/pharmacodynamic (PK/PD) properties* in blue, and *promising efficacy/safety* in green. PVI indicates pulmonary vein isolation. Figure created with BioRender.com.

The routine treatment strategy for patients with AF includes anticoagulants, rate control, and rhythm control.^74^ It is widely accepted that obesity can alter the pharmacokinetic and pharmacodynamic properties of drugs, especially volume of distribution and elimination.^75^ Experimental studies summarized in section Electrical Remodeling also demonstrate that obesity can alter expression and activity of common targets for AADs (eg, voltage‐gated sodium channels). Furthermore, the lipophilicity of the drug is postulated to alter its effectiveness in obese patients with AF. Finally, there is clear evidence that obesity can affect outcomes after ablation, such as increased AF recurrence. Thus, the effect of obesity on AF treatment (Figure 4) is an important consideration in formulating therapeutic strategies and requires further research.

## THE EFFECT OF OBESITY ON ANTICOAGULANTS

Patients with AF are routinely given anticoagulants to prevent ischemic stroke.^74^ However, there is limited evidence for the use of anticoagulants in patients with a weight >120 kg or a BMI >40 kg/m^2^. A study examining the effectiveness of warfarin, a common anticoagulant that inhibits vitamin K–dependent coagulation proteins, found that the dose required to achieve international normalized ratio within the therapeutic range was positively associated with body weight.^76^ A meta‐analysis confirmed these findings and recommended that obese and morbidly obese patients may benefit from an increase in warfarin dose of 30% to 50%.^77^ Plasma vitamin K is positively correlated with triglyceride concentrations and, therefore, warfarin dose.^76^ This may explain why the optimal warfarin dose increases with weight. In addition, obese and morbidly obese patients prescribed warfarin needed a longer median time to achieve a therapeutic international normalized ratio, a higher average daily dose, and mean discharge dose than healthy‐weight patients.^78^ Together, this evidence indicates that BMI status should be considered during the initiation of warfarin therapy.

Direct oral anticoagulants (DOACs) are increasingly recommended over warfarin for stroke prevention in patients with AF.^74^ Russo et al^15^ found that DOAC plasma concentration was not associated with weight. A study on rivaroxaban demonstrated that weight alone has little impact on drug pharmacokinetics.^79^ The study by Wasan et al^80^ refuted this result, reporting peak plasma concentration and half‐life of apixaban were significantly reduced in morbidly obese patients. Wasan et al also showed that the rate of increase in the drug level was inversely correlated with body weight from baseline to 2 hours. Conversely, from 2 to 4 hours, a positive trend between body weight and the rate of increase in apixaban level was shown, although this was insignificant when comparing groups.

Evidence about the impact of obesity on safety and the effectiveness of anticoagulants is limited. A systematic review including 13 studies found no indication that obesity is related to inferior DOAC effectiveness or safety.^72^ These findings support the recommendation that a standard fixed dose of DOACs is adequate for healthy‐weight and obese patients. However, further research is required to conclusively determine optimal dosing in morbidly obese patients.

Compared with warfarin, DOACs do not require laboratory monitoring to confirm the international normalized ratio, providing easier maintenance. The ENGAGE AF‐TIMI 48 trial has shown that DOACs and warfarin were similar in efficacy and safety.^69^ On the contrary, a meta‐analysis including 9 studies demonstrated that DOACs were related to significantly lower risk of stroke and major bleeding compared with warfarin, irrespective of weight.^81^ The meta‐analysis also showed that overweight patients treated with a DOAC had a lower risk of all‐cause death. Another meta‐analysis including 10 studies with 89 494 morbidly obese patients with AF showed that the risk of stroke and major bleeding in the DOAC group was lower compared with the warfarin group.^82^ On subgroup analysis, rivaroxaban and apixaban demonstrated better performance in effectiveness and safety than warfarin, whereas dabigatran only showed superiority in the safety outcome over warfarin. In conclusion, the effectiveness and safety of DOACs in overweight and obese patients is equal to or surpasses that of warfarin. Considering strict laboratory monitoring of warfarin, DOACs are a better, lower‐maintenance choice for obese patients with AF to prevent stroke. However, large‐scale randomized clinical trials are needed to prove this.

## THE EFFECT OF OBESITY ON RATE CONTROL

The therapeutic goal of rate control is to regulate heart rate to ≤80 beats per minute at rest.^74^ β‐Blockers are used to slow the heart rate in patients with AF. Lipophilicity of a drug can alter its pharmacokinetics in obese patients. Sotalol, a markedly hydrophilic β‐blocker, showed similar pharmacokinetic parameters in obese and lean patients, whereas lipophilic β‐blocker propranolol had a lower volume of distribution in obese patients.^14^ Total volume of distribution at steady state for labetalol and nebivolol (lipophilic β‐blockers) was larger in obese than in healthy‐weight subjects, and positively related to weight.^83^ However, when accounting for body weight, volume of distribution (per kilogram) is slightly but not significantly smaller in obese patients. These results suggest lipophilic β‐blockers preferentially diffuse into lean tissue over adipose tissue,^83^ in contrast to other lipophilic drugs that commonly show increased distribution volume and prolonged elimination half‐life in obese patients.^75^ Thus, additional factors to lipid solubility are possibly involved in β‐blockers' pharmacokinetics in obese patients. Changes in heart rate, blood pressure, and cardiac output in response to nebivolol and labetalol were similar in obese and nonobese volunteers, respectively, who received comparable doses.^83^

A study on diltiazem, a nondihydropyridine calcium channel blocker for rate control management of AF with a rapid ventricular response, also confirmed the impact of obesity on rate control drugs.^84^ In this study, obese patients receiving a weight‐based dose were significantly more likely to achieve a heart rate <100 beats per minute than those administered a fixed dose. In addition, the distribution and clearance of digoxin, a positive ionotropic cardiac glycoside, were seemingly unaffected by obesity.^85^ However, no studies to date report on digoxin effectiveness in obese versus healthy‐weight patients with AF.

## THE EFFECT OF OBESITY ON RHYTHM CONTROL

### Pharmacologic Rhythm Control Strategies

Class I AADs are commonly used for rhythm control by inhibiting sodium channels to reduce cardiac excitability and abnormal automaticity. Class I AADs primarily block fast sodium channels, which are responsible for rapid depolarization of phase 0 of the cardiac action potential. Ornelas‐Loredo et al^12^ found that nonresponse to class I AADs in obese patients was greater than in healthy‐weight patients (30% [obese] versus 6% [nonobese]; *P*=0.01). For every 2.5‐unit increment of BMI, there was a reduced effectiveness of class I AADs in patients with obesity. Flecainide, a class Ic AAD, reduces peak sodium current by inhibiting voltage‐gated sodium channels. Given that sodium current is reduced in HFD mouse atrial myocytes,^41^ it is difficult to explain the reduced effectiveness of flecainide in obese patients. Our recently published work uncovered increased atrial effectiveness of flecainide in healthy myocardium, likely driven by altered biophysical properties compared with ventricles.^86^ The effect of obesity on inter‐chamber differences in sodium current density and β‐subunit expression may elucidate mechanisms by which flecainide shows reduced efficacy in AF.

Mexiletine, a class Ib AAD to preferentially inhibit late sodium current, showed higher efficacy in reducing AF inducibility in obese mice in vivo compared with saline control.^13^ In HFD mice, late sodium current is increased, which can lead to prolonged APD, repolarization dispersion, and susceptibility to arrhythmogenic afterdepolarizations.^13^ The therapeutic effect of mexiletine in obesity‐mediated AF is suggested to be mediated via decreased late sodium current, thus reducing APD and afterdepolarization risk. The mechanism of mexiletine selectivity for late sodium current over peak current is unknown.

Class III AADs block potassium channels, leading to increased APD and ERP, increased cardiac wavelength and prevention of reentry. Amiodarone is a class III AAD used for the treatment of AF and ventricular arrhythmias.^74^ Amiodarone and its metabolite, N‐desethylamiodarone, have high lipophilicity, and particularly accumulate in adipose tissue.^87^ Fukuchi et al^88^ provided evidence that amiodarone clearance was affected by BMI, reduced by 22.3% when BMI was >25 kg/m^2^, thus resulting in significantly higher serum concentration in obese patients. Reduced expression of cytochrome P450 enzymes in HFD mice was demonstrated,^89^ providing a likely mechanism for reduced elimination of amiodarone. Reduced hepatic metabolism and increased levels of amiodarone‐binding proteins may contribute to altered amiodarone pharmacokinetics in obesity.^89^

However, Ornelas‐Loredo et al^12^ showed that the therapeutic response to class III AADs, including amiodarone, is similar between obese and healthy‐weight patients. In the obese patient group, class I AAD nonresponse was greater than class III. To further compare the effect of obesity on different AAD class efficacy, AF burden was measured before and after treatment of flecainide (class I) and sotalol (class III) AADs, respectively, in pacing‐induced AF HFD mice. The percentage reduction in AF burden was greater for sotalol compared with flecainide.^12^ These results indicate that obesity impairs the class I AAD response but has little effect on the class III response. Although there is evidence for changes in expression of proteins targeted by these therapeutics in HFD mice,^13^, ^41^ further work is required to determine the mechanisms driving the reduced effectiveness. European Society of Cardiology guidelines recommend class I AADs over class III AADs for patients with AF,^74^ with no mention of specific recommendations for obese patients with AF. On the basis of the current evidence outlined in this review, we would challenge this guidance for the obese population with AF, because class III AADs may show higher efficacy in the obese patient subgroup over class I. Further randomized clinical trials are required to establish clear AAD guidance in obese patients, given that this evidence is based mainly on observational clinical studies.

### Cardioversion and Ablation Rhythm Control Strategies

Direct cardioversion is commonly used as a means of converting AF to sinus rhythm. Both pharmacologic and electrical cardioversion failure was demonstrated to be more frequent in obese patients.^90^, ^91^ Voskoboinik et al^91^ suggested that the use of more powerful defibrillators may increase success rates of cardioversion in morbidly obese patients. The probable reason may be that obese patients have more EAT, intrathoracic fat, and visceral adipose tissue, which block energy delivery to the heart.^26^ Indeed, EAT was an independent predictor of electrical cardioversion success in patients with nonvalvular persistent AF, where AF recurrence associated with significantly greater EAT thickness.^24^

Catheter ablation involves burning or freezing cardiac tissue, primarily around the pulmonary veins, to prevent aberrant electrical activity. Catheter ablation can provide an effective treatment for AF.^74^ The impact of obesity on catheter ablation, including outcomes, AF recurrence, and complications, has been extensively evaluated. Obese patients are less likely to have arrhythmia‐free survival after ablation and are therefore more likely to undergo repeated procedures.^6^, ^26^ Obese patients with AF may demonstrate altered ectopic foci compared with nonobese patients with AF, rendering standard ablation targets, such as pulmonary vein isolation, inadequate. Hu et al^92^ performed driver ablation or conventional pulmonary vein isolation ablation in obese patients with AF and demonstrated that driver ablation improved long‐term outcomes in obese patients. Obesity was related to increased driver complexity, especially in the LA posterior wall. Catheter ablation targeting the posterior LA wall had a higher rate of termination of AF in obese compared with healthy‐weight patients with AF.^92^ Considering that obese patients with AF show an increase in EAT volume, especially around the posterior LA,^18^ EAT may provide a substrate perpetuating AF, but also an ablation target. Hybrid convergent ablation, combining minimally invasive surgical (epicardial) and catheter (endocardial) ablation, is more effective than endocardial catheter ablation.^93^ Hybrid convergent ablation has an advantage of ablating the target structures of interest,^93^ including EAT and ganglionated plexi, which may serve as drivers of AF.

Although the most recent meta‐analysis reported equal efficacy of radiofrequency and cyroballoon catheter ablation in patients with persistent AF,^94^ cyroballoon ablation may demonstrate superior efficacy in obese patients with AF.^95^ Obesity was also independently related to higher ionizing radiation exposure during ablation,^96^ elevated in‐hospital complications, increased mortality, longer length of stay, and higher hospital costs in patients with AF after ablation.^97^ These findings provide a strong argument for a negative impact of obesity on outcomes in AF, despite the obesity paradox previously described.

Overall, ablation strategies for obese patients with AF must consider identifying and targeting sources of ectopic activity, alternative methods of ablation delivery and accurate dosing to optimize patient outcomes.

### The Effect of Obesity on AF After Cardiac Surgery

Postoperative AF (POAF) is the most common complication after cardiac surgery.^98^ The occurrence of POAF is multifactorial, and obesity is recognized as an independent risk factor.^98^ Recently, meta‐analyses have found that EAT was associated with POAF.^25^ The underlying mechanism of POAF is not fully elaborated. Possible mechanisms may include pericardial inflammation, including EAT‐mediated interleukin‐6 and MCP‐1 (monocyte chemoattractant protein‐1) signaling,^99^ and autonomic imbalance after cardiac surgery.^100^ EAT has an abundance of ganglionic plexi,^43^ which may contribute to postsurgery autonomic imbalance and initiating POAF.^52^ In obesity, EAT also undergoes disequilibrium between excessive oxidative stress and protective adipokine levels,^20^, ^43^ promoting POAF susceptibility. Obesity‐mediated structural remodeling, including increased cardiac fibrosis, left ventricular hypertrophy, and LA dilatation, may also trigger POAF because they are independent risk factors of AF.^37^

## CONCLUSIONS

Obesity mediates AF by structural and electrical remodeling, including adiposity, neurohormonal changes, inflammation, fibrosis, and oxidative stress. Obesity can also affect AF treatment success through alterations in drug pharmacokinetics and target expression or function. On the basis of the evidence presented in this review, health guidelines should consider recommending DOAC and class III AADs for obese patients with AF because they retain similar efficacy in obesity. Obese patients with AF are more likely to experience inferior catheter ablation outcomes, comorbidities, and POAF. Although the obesity paradox has been postulated in AF, weight loss is a demonstrated beneficial measure in reducing AF burden and postablation recurrence. In addition, EAT, a key mediator of obesity‐linked AF, provides a promising therapeutic target, both pharmacologically and in ablation. Future studies should focus on improving understanding of the interactions between obesity, AF, and AF treatment. Both early stratification and personalized treatment must be prioritized to improve patient outcomes in obesity‐mediated AF.

## Sources of Funding

The authors are supported by the British Heart Foundation (PG/17/55/33087, RG/17/15/33106, FS/19/12/342040; FS/PhD/22/29309); British Heart Foundation Accelerator Award to the Institute of Cardiovascular Sciences, University of Birmingham; Wellcome Trust (109604/Z/15/Z; 221650/Z/20/Z); MRC AIM DTP (MR/W007002/1).

## Disclosures

None.
